# Synthesis-Dependent Strand Annealing in Meiosis

**DOI:** 10.1371/journal.pbio.0050299

**Published:** 2007-11-06

**Authors:** Melissa S McMahill, Caroline W Sham, Douglas K Bishop

**Affiliations:** 1 Committee on Genetics, University of Chicago, Chicago, Illinois, United States of America; 2 Department of Radiation and Cellular Oncology, University of Chicago, Chicago, Illinois, United States of America; 3 Department of Molecular Genetics and Cell Biology, University of Chicago, Chicago, Illinois, United States of America; National Cancer Institute, United States of America

## Abstract

Recent studies led to the proposal that meiotic gene conversion can result after transient engagement of the donor chromatid and subsequent DNA synthesis-dependent strand annealing (SDSA). Double Holliday junction (dHJ) intermediates were previously proposed to form both reciprocal crossover recombinants (COs) and noncrossover recombinants (NCOs); however, dHJs are now thought to give rise mainly to COs, with SDSA forming most or all NCOs. To test this model in *Saccharomyces cerevisiae*, we constructed a random spore system in which it is possible to identify a subset of NCO recombinants that can readily be accounted for by SDSA, but not by dHJ-mediated recombination. The diagnostic class of recombinants is one in which two markers on opposite sides of a double-strand break site are converted, without conversion of an intervening heterologous insertion located on the donor chromatid. This diagnostic class represents 26% of selected NCO recombinants. Tetrad analysis using the same markers provided additional evidence that SDSA is a major pathway for NCO gene conversion in meiosis.

## Author Summary

In organisms that reproduce sexually, sex cells (gametes) are produced by the specialized cell division called meiosis, which halves the number of chromosomes from two sets (diploid) to one (haploid). During meiosis, homologous DNA molecules exchange genetic material in a process called homologous recombination, thereby contributing to genetic diversity. In addition, a subset of recombinants, called crossovers, creates connections between chromosomes that are required for those chromosomes to be accurately segregated. Accurate segregation ensures that gametes contain one and only one copy of each chromosome. Recombination is initiated by chromosome breakage. A regulatory process then selects a subset of breaks to be healed by a mechanism that forms crossover recombinants. Many of the remaining breaks are healed to form so-called “noncrossover” recombinants (also referred to as “gene conversions”). Until recently, it was thought that crossovers and noncrossovers were formed by nearly identical pathways; which type of recombinant arose was thought to depend on how the last enzyme in the pathway attacked the last DNA intermediate. However, more recent observations suggested that noncrossover recombinants might arise by a mechanism involving less-stable intermediates than those required to make crossovers. In the present work, a yeast strain was constructed that allowed the detection of a genetic signature of such unstable recombination intermediates. This strain provided evidence that meiotic crossovers and noncrossovers do indeed form by quite different mechanisms.

## Abstract 3

New evidence suggests that meiotic noncrossover recombination can occur without a Holliday junction intermediate.

## Introduction

Homologous recombination is essential for meiosis, the cellular division process specific to gametogenesis. The reduction in ploidy that occurs during meiosis is necessary for sexual reproduction and is achieved by a single round of DNA replication followed by two rounds of chromosome segregation. Reductional segregation is distinguished from mitotic or equational segregation in that sister centromeres remain together during the metaphase-to-anaphase transition of the former, but separate to opposite poles during the latter. For the two pairs to be accurately segregated, they must first become physically connected to one another. Homologous recombination forms physical connections called chiasmata between the replicated pairs of homologs (reviewed in [1]). In addition to being required for reductional chromosome segregation, meiotic recombination makes a major contribution to genetic diversity by generating both new alleles and allele combinations.

There are two major classes of meiotic recombination, both of which arise from a common precursor, a DNA double-strand break (DSB). If chromosome arms on opposite sides of the recombination initiation site swap partners, the event is designated a reciprocal crossover (CO). If the original configuration of chromosome arms is retained, the event is designated a noncrossover (NCO). Both CO and NCO events can result in a type of non-Mendelian segregation, called gene conversion, of heterozygous markers near the recombination initiation site. Only COs form the chiasmata needed for chromosome segregation, yet approximately two-thirds of recombination events in budding yeast are NCOs. The proportion of NCOs may be higher in mammals [2], as the number of total recombination events, estimated from Rad51/Dmc1 foci, dramatically exceeds that of CO-specific events. Understanding the mechanisms that give rise to both CO and NCO recombinants is critical to understanding how the decision is made to convert a meiotic DSB into a CO or an NCO recombinant.

**Figure 1:**
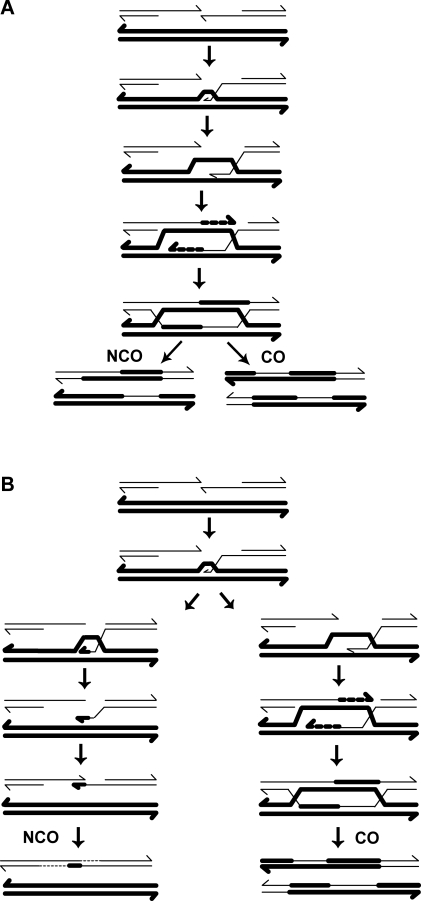
Models for Meiotic Recombination (A) The dHJ model [6]. Recombination is initiated by a DSB. Ends are resected to form 3′ single-stranded tails. One end invades the intact homolog to form a D-loop that is enlarged by extension of the invading end by DNA synthesis using the intact strand as template. End extension enlarges the D-loop, making it possible for the second end to anneal to the D-loop. After the second end anneals, repair synthesis and ligation forms a dHJ. CO and NCO products arise from the relative orientation of two HJ resolution events. (B) The early crossover decision model [25,26]. Ends are resected, and one DSB end forms a D-loop with its homolog and is then extended by DNA synthesis, as in the dHJ model. If a CO is to be formed, events follow those as in (A). If an NCO is to be formed, after the end is extended, the D-loop is disrupted by displacement of the extended end. The displaced end then undergoes synthesis-dependent strand annealing (SDSA; see text): Repair synthesis and ligation forms an NCO recombinant. As drawn here, SDSA forms only NCOs. A derivative of this model showing how a CO can be associated with SDSA is provided in Figure S3.

Tetrad analysis in fungi showed that gene conversion of a marker is frequently associated with reciprocal exchange of flanking markers [3,4]. This association was neatly accounted for by Robin Holliday's proposal that recombination involved an intermediate in which only two of the four single DNA strands were exchanged [5]. Depending on which single strands are nicked, the Holliday junction (HJ) intermediate can be resolved to form either a CO or a NCO. The subsequent evolution of models for recombination retained the HJ as a common intermediate explaining the origin of COs and NCOs. This feature was retained even when Szostak et al. proposed the Double-strand Break Repair model [6] (Figure 1A), in which two HJs form and are resolved during each recombination event. Hereafter this model will be referred to as the “dHJ model” (double Holliday Junction model).

With respect to the meiotic recombination mechanism in budding yeast, many of the predictions of the dHJ model have been fulfilled (for reviews see [7–9]): Meiotic recombination is initiated by DNA DSBs [10,11]. The breaks are processed by resection of the 5′ ends, resulting in a pair of 3′ single-stranded overhanging ends [10,12]. Homologous joint molecules are formed when these ends invade an intact donor chromosome, creating hybrid DNA with complementary strands from the donor [13,14]. the 3′ invading ends in the joint molecule serve as primers for repair synthesis [10]. The ends of newly synthesized segments are ligated to the resected 5′ ends, forming a specific type of joint molecule, the predicted dHJ [15–19]. According to the dHJ model, the dHJ is converted into recombination products via the action of a nicking endonuclease. The orientation of one HJ resolution event relative to the other determines whether the dHJ intermediate will give rise to a CO or an NCO.

Although several of its key predictions were fulfilled, some observations are not compatible with the original dHJ model. Of interest to the current work are studies suggesting that this model does not account for the observed properties of NCO recombinants. The dHJ model predicts a specific configuration of hybrid DNA regions relative to the site of the initiating DSB: one of the two recombining chromatids is predicted to have a heteroduplex patch to the left of the initiating DSB, whereas the other recombining chromatid is predicted to have a heteroduplex patch to the right. Several studies designed to test this prediction found that the expected bi-directional configuration of heteroduplex segments was rare [20–24]. Instead, there was a prevalence of events in which evidence of heteroduplex DNA was found only on one side of the initiating DSB. Other unexpected configurations of heteroduplexes were also seen. Of particular note was a class of recombinants with two tracts of heteroduplex, both on the same chromatid [21,24]. The original dHJ model does not account for this type of recombinant.

In addition to heteroduplex DNA configurations that did not match predictions of the original dHJ model, studies of mutants displaying partial defects in meiotic recombination challenged the notion that CO and NCO recombinants result from alternative orientations of HJ resolution (reviewed in [25]). Mutations that cause specific defects in CO formation were found to result either in accumulation of dHJs [26] or in preventing the appearance of dHJs [27]. One such mutation is *zip1* [27,28]. These results suggested that the dHJs seen on 2D gels are predominantly intermediates to the formation of COs, but not NCOs. Differences in the properties and timing of appearance of COs and NCOs led Allers and Lichten [26] to propose that a major fraction of NCOs results not from a mechanism involving a ligated dHJ, but rather from synthesis-dependent strand annealing (SDSA). In this model, which we have referred to as the “early CO decision” model (Figure 1B) [25], a meiotic DSB is designated to become a CO or NCO before the formation of a ligated dHJ.

SDSA is a mechanism in which homology-mediated repair of DSBs occurs without formation and resolution of ligated HJs. Resnick proposed the earliest model with the critical features of SDSA [29], although it did not receive its current name until later [30]. During SDSA, repair of a DSB is achieved by invasion of an overhanging 3′ end into the intact donor chromatid. The joint formed by invasion may be subject to mismatch repair, leading to shortening of the invading end. Following this opportunity for mismatch excision, repair synthesis can extend the invading end past the site of the DSB. Once the end is extended, disruption of the joint occurs. The extended end can then anneal with its partner. The product of annealing is then converted to an intact duplex by repair synthesis and ligation. SDSA differs from models that involve HJ intermediates in that its simplest version accounts only for NCO products, although models for SDSA giving rise to CO products have been suggested [7,31–33]. Versions of the SDSA model were proposed to explain properties of budding yeast mating-type conversion that did not fit well with the HJ intermediate model, including the fact that mating-type conversion is not associated with crossing over [34–37].

Critical evidence for SDSA was obtained by induction of DSBs by P-element excision in mitotic cells of the *Drosophila* germ line [30,38,39]. A key aspect of these studies was the demonstration that a recipient chromatid could collect sequences from more than one donor locus during a DSB repair event [30]. This finding implied that end extension at one locus can be followed by the disruption of the homologous joint prior to the formation of a second homology-mediated connection between donor and recipient molecules [30]. In addition, the ability of a broken DNA molecule to collect sequences from separated donor loci was shown in mitotic budding yeast using plasmids or endonuclease induction of chromosomal events [33,40–42]. Other studies provided additional support for the conclusion that SDSA is a predominant mechanism for mitotic NCO recombination in budding yeast and other organisms (reviewed in [7], see also [43–45]). Furthermore, SDSA provides a reasonable explanation for the patterns of heteroduplex DNA seen among NCOs in budding yeast meiosis.

Although several observations are consistent with the possibility that SDSA contributes to NCO recombination in meiosis, there have been no specific tests of this hypothesis. To address this issue, we created a recombination system that provides evidence for SDSA in a manner analogous to the previously described mitotic systems [30,33], in which recipient ends collect sequences from separated donor loci. Our results provide evidence that SDSA is an important mechanism of NCO recombination in meiosis.

## Results/Discussion

### Experimental System

**Figure 2:**
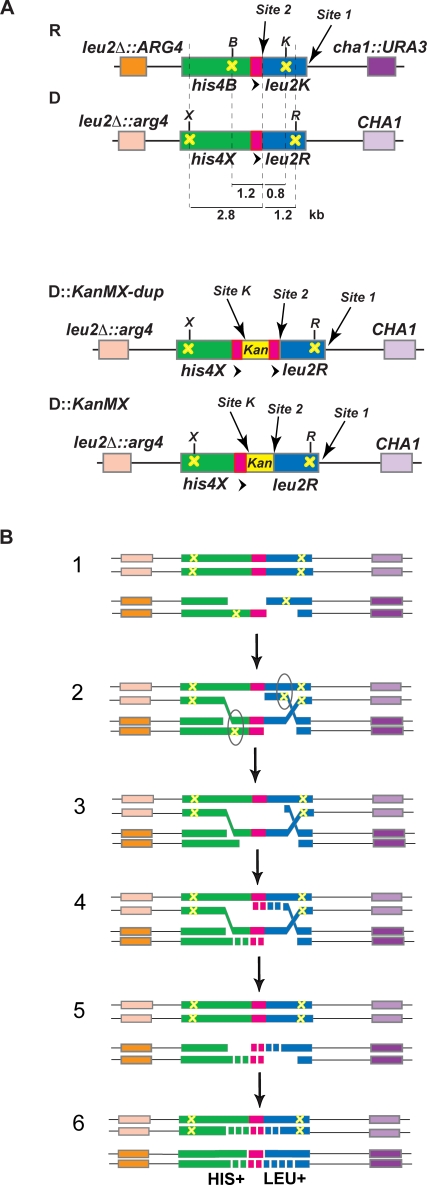
The “Ends-Apart” Recombination System (A) *D* (“Donor”) and *R* (“Recipient”) contain heteroalleles of *his4* and *leu2* genes; vertical lines through the heteroalleles indicate the positions of the restriction site fill-in mutations. Breaksites 1, 2, and K (the breaksite in the *KanMX* cassette) are indicated. The intensities of these breaksites are: site 1 = 12%, site 2 = 3%, and site K = 1.5%. Distances from site 2 (the breaksite of interest to this study) to each of the heteroallelic mutations are shown. *D::KanMX-dup* contains a heterologous *KanMX* cassette inserted between the two heteroalleles, and also contains a duplicated segment of the 5′ end of *leu2*; the duplicated segment is shown as a red box over an arrowhead. *D::KanMX* is identical to *D::KanMX-dup* but does not contain the duplication. Site 2 is located ≤100 bp away from the *KanMX* insertion point. In *R/D* diploids, two heterozygous markers flank *HIS4::LEU2;* an *ARG4* cassette (peach/orange) inserted at the *LEU2* locus, replacing the *LEU2* coding sequence and a *URA3* cassette (lavender/purple) inserted at the *CHA1* locus. (B) During meiosis, after a break at site 2 on the *R* chromosome, wild-type sequences from the *D* chromosome must be used to repair the resected regions of *R* that had carried the *his4B* and *leu2K* mutations. Mutations are shown by yellow x's. (1) The *R* chromatid is cleaved at site 2, and 5′ ends are resected, leaving 3′ overhanging tails. (2) 3′ tails invade the homolog forming a D-loop, and the mismatch repair (MMR) machinery recognizes mismatches (circled). (3) Mismatches are removed by the MMR machinery. (4) Repair synthesis uses *D* chromatid information to repair the *R* molecule to His^+^Leu^+^ prototrophy. (5) Invading ends are displaced and anneal. (6) Repair synthesis and ligation for a *HIS4^+^LEU2^+^* chromatid.

A reporter strain was constructed to test the SDSA model for meiotic NCO recombination. The reporter strain carries a configuration of markers designed to allow the identification of a diagnostic class of NCO recombinants whose origin can be simply explained by SDSA but not by the dHJ model. This diagnostic class is one in which two markers on opposite sides of a DSB are converted, without conversion of an intervening heterologous insertion on the donor chromatid. The system is designed to provide relevant data by analysis of random spores rather than of tetrads. The advantage of random spore analysis is that a much larger number of relevant recombination events can be scored than would be possible by tetrad analysis. Accompanying tetrad data provide evidence that the recombination events selected in the random spore analysis are representative of typical gene conversion events. What follows is a description of the reporter system that we designate the “ends apart” system (Figure 2).

The ends-apart system uses a cassette containing a functional copy of the *LEU2* gene inserted downstream of the *HIS4* locus (Figure 2A) [11,46]. The *HIS4::LEU2* construct is a well-characterized recombination hotspot ([27] and references therein). Hotspot activity at *HIS4::LEU2* results from two strong DSB sites, one downstream of *LEU2* (site 1) and, more importantly for this system, a second break site (site 2) in between *HIS4* and *LEU2*. Two derivatives of the standard *HIS4::LEU2* locus exist *(his4B::leu2K* and *his4X::leu2R),* each carrying a single mutation in *HIS4* and a single mutation in *LEU2*. These mutations are 4-bp insertions that cause frame shifts; haploid strains carrying these derivatives are auxotrophic for histidine and leucine. As shown in Figure 2A, the mutations carried by *his4B::leu2K* are relatively close to site 2 (1.2 and 0.8 kb, respectively). This derivative is termed “Recipient”, or “*R*,” because its configuration of markers is such that the majority of events yielding an NCO chromatid with the two functional alleles required to satisfy the selection will be those in which the *R* chromatid is the “recipient” of genetic information. The mutations carried by the second derivative (*his4X::leu2R*) are both farther from site 2 than are those on *R* (2.8 and 1.2 kb, respectively). This derivative is called “Donor”, or “*D*,” because the markers it carries make it most likely to be the donor chromatid during the events that give rise to the selected recombinants. The configuration of markers is such that a single DSB at site 2 on the *R* chromatid in an *R/D* heterozygote can yield a *HIS4^+^::LEU2^+^* chromatid via end-directed mismatch repair or by extension of invading ends past the DSB proximal marked site but not the DSB distal site (reviewed in [7]; Figure 2B). Conversion of these break-proximal markers will yield an *R* chromatid carrying *HIS4^+^::LEU2^+^*. These His^+^Leu^+^ products will be referred to as “double prototrophs.” The haploid spores that inherit such *HIS4^+^::LEU2^+^* chromatids can be selected by germination and growth on medium lacking both histidine and leucine. The *HIS4::LEU2* region of *R/D* heterozygotes is flanked by heterozygous markers that allow noncrossover recombinants to be distinguished from crossover recombinants. The *D* chromatid also carries DSB site 2, but the strong tendency of DNA ends to impose directionality on mismatch repair events in favor on the unbroken chromatid dictates that single breaks on *D* will only very rarely lead to the formation of double prototrophs.

**Figure 3:**
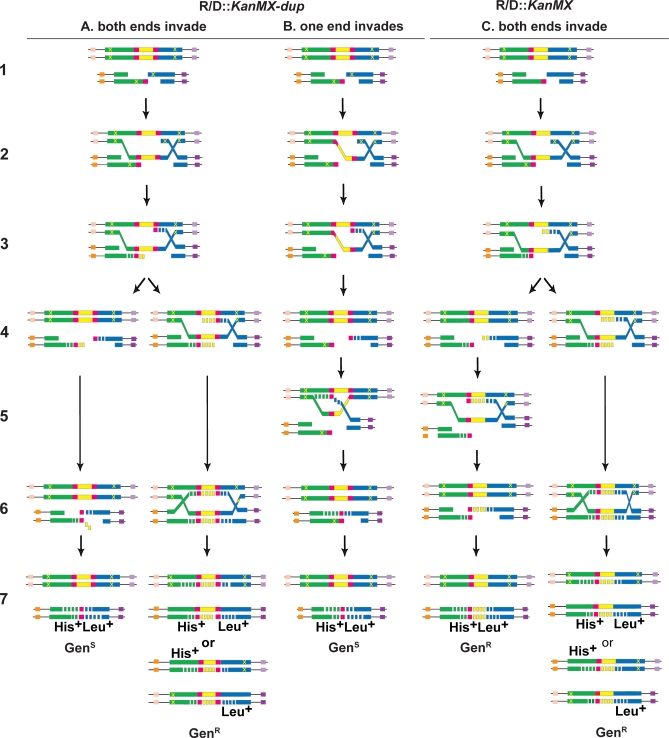
Models for Generation of *HIS4^+^ LEU2^+^* Recombinants via a Single DSB in the Ends-Apart System Colors represent the same markers as described in Figure 2. (A) Both ends formed by a DSB on *R* invade *R/D*::*KanMX-dup*. (B) Only one of the two ends invades *R/D*::*KanMX-dup*. In (A) and (B), the duplicated segment allows for annealing of two ends, each acquiring sequences from different copies of the duplication, thus making it possible to generate *HIS4^+^LEU2^+^* recombinants that lack *KanMX*. (C) Creation of *HIS4^+^LEU2^+^* recombinants in *R/D*::*KanMX*. Resolution of NCOs by the SDSA mechanism is only expected to give recombinants that contain *KanMX*. The basic events common to all three mechanisms (A–C) are: (1) The DSB site between *his4* and *leu2* on *R* is cleaved. (2) End invasion. (3) Mismatch repair at the site of *his4B* and *leu2K* and repair synthesis to extend ends into or through the duplicated region (in *R/D*::*KanMX-dup*), but not all the way across the *KanMX* gene. (4) D-loop disruption. (5) Opportunity for annealing. Ends that contain complementary sequences can anneal. Ends than cannot anneal may reinvade and undergo further extension until sequences complementary to the partner end are added. (6) Strand annealing of disrupted ends. dHJ formation for ends that remain in D-loops. (7) Repair synthesis and ligation, or HJ resolution.

In the ends-apart system, a single meiotic initiation event could yield a double-prototrophic haploid recombinant from an *R/D* diploid by more than one mechanism. In one scenario, bi-heteroduplex tracts could form; i.e., both DNA ends generated by a site 2 DSB on *R* could invade *D* to form two hybrid DNA segments (as in the canonical dHJ model). Both such segments may incorporate a break-proximal marker into hybrid DNA. Efficient correction of the mismatched sites would then be expected to occur favoring the sequence on the *D* strand. The repair synthesis that follows will copy homologous sequences from the donor chromatid and may extend past the site of the break. Following repair synthesis, the homologous joint may then be resolved in one of three ways. First, displacement of both invading strands from the joint may lead to SDSA, creating a chromatid that carries *HIS4^+^::LEU2^+^*. Secondly, additional repair synthesis may be followed by ligation of ends to form a dHJ. Such a ligated junction could then be resolved by structure-specific endonucleases as in the canonical dHJ model or by topoisomerase activity, (as proposed by Gilbertson and Stahl in 1996 [24]). Only NCOs formed from dHJ intermediates are expected to place both *HIS4^+^* and *LEU2^+^* recombinants on the same chromatid as required to satisfy the selection for double prototrophs among haploid spores; resolution of a dHJ to form a CO is expected to place the two prototrophic alleles on different recombinant strands (Figure 3). A third scenario that could yield double prototrophs from a single break would involve invasion of only one of the two ends. In this case, invasion and subsequent mismatch repair within the hybrid DNA region could be followed by the formation of a synthesis tract extending not just past site 2, but also past the opposite break-proximal marked site. Disruption of the joint containing this twice-extended end could lead to annealing and formation of the selected *HIS4::LEU2* recombinant via SDSA.

The three scenarios described above illustrate that an *R/D* diploid can form a *HIS4^+^::LEU2^+^* haploid spore either by transient interaction (of one or both ends) or by the stable interaction of both ends, yielding a dHJ intermediate. To distinguish between these mechanisms, we created a class of recombinants that are diagnostic for SDSA. This was accomplished by additional modification of the *D* chromosome. One such modified construct is called *D::KanMX-dup* (Figure 2A). The insertion in *D::KanMX-dup* has two elements. The first element is a directly oriented duplication of a 300-bp fragment from the 5′ end of the *leu2* cassette. The duplicated segment ends at a region corresponding to site 2. The second element, which separates the two copies of the duplicated region of the *leu2* cassette, is a 1.5-kb *KanMX* cassette [47] conferring resistance to the fungicide geneticin thereby giving a geneticin-resistant (Gen^R^) phenotype. In an *R/D*::*KanMX-dup* diploid, the position of the duplicated segment relative to the *KanMX* cassette makes it possible to form an extended end with sufficient homology to anneal back to the partner end. Formation of such “annealing competent” intermediates could occur in either of two ways (Figure 3A and 3B). First, both ends could invade (Figure 3A) and, via mismatch repair and synthesis, collect sequences needed to form *HIS4^+^* and *LEU2^+^*_,_ with one of the ends extending past site 2 before the disruption of both joints. Alternatively, two rounds of invasion by a single end (Figure 3B) may occur. The first invasion results in mismatch resection and extension across the break site into the proximal copy of the duplicated sequence. The joint is then disrupted, and the extended end reinvades the distal copy of the duplicated sequence. If additional end extension proceeds past the break-proximal marked site, the strand generated may form *HIS4^+^::LEU2^+^* recombinants via SDSA. Both the two-end and one-end mechanisms described require dissociation of hybrid DNA as proposed for SDSA. SDSA could also yield Gen^R^ products (Figure 3A), but this class can also be accounted for by the mechanism involving a dHJ. Thus, the ascospore phenotype of the class diagnostic for SDSA is His^+^Leu^+^Gen^S^.

In the ends-apart system, the ability to recover His^+^Leu^+^Gen^S^ recombinants via SDSA is predicted to depend on the use of both copies of the duplicated 5′*-leu2* segment as templates for end-extension during the recombination event. This prediction was tested by construction of a second modified *D* chromosome lacking the duplicated copy of 5′*'-leu2*, called *D::KanMX* (Figure 2). Dependence of the yield of the His^+^Leu^+^Gen^S^ on the presence of the duplicated segment is taken as evidence that SDSA contributes to this class. In Figure 3C, both ends of the *R* chromosome are shown invading *D::KanMX,* but the invasion of either one or two ends is predicted to result in the same His^+^Leu^+^Gen^R^ product.

A final feature of the system is a pair of linked heterozygous flanking markers located on either side of the *HIS4::LEU2* locus, far enough away from site 2 that they will not influence conversion tracts*.* These markers make it possible to determine if a particular His^+^Leu^+^ recombinant is a CO or an NCO product. The *R* chromosome carries two marker insertions that the *D* constructs lack: (1) an insertion of a cassette carrying the *ARG4* gene replaces the entire coding region of the normal *LEU2* locus, which is located 23 kb (11 cM) centromere-proximal to *HIS4::LEU2*; and (2) an insertion of a cassette carrying the *URA3* gene at the *CHA1* locus, which is located 40 kb (34 cM) centromere-distal to *HIS4::LEU2* .

The configuration of markers in the ends-apart system was designed to allow the detection of the signature of SDSA while avoiding interaction of ectopic or heterologous sequences. *KanMX* insertion was placed at the site of a DSB to allow the selected events to occur by a mechanism very similar to that which would occur in the absence of any heterology. This is important because neighboring heterologies can alter the properties of a homologous recombination event [48,49]. Comparison of the two strains used to test for SDSA with a control strain lacking the heterologous insertion showed that all three strains yield single and double prototrophs at equivalent frequencies (Table 1). This finding provides evidence that the mechanism generating the selected recombinants is not substantially altered by the presence of the heterologous *KanMX* insertion and is likely to be representative of normal allelic recombination.

**Table 1:**
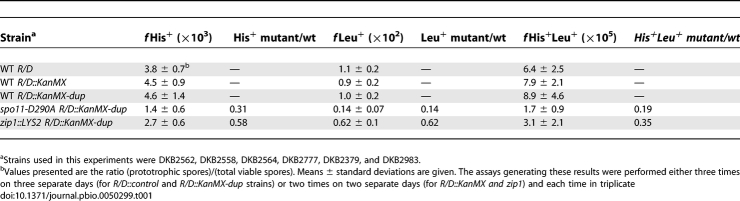
Mean Prototroph Frequencies from Random Spores

### Evidence That NCO Meiotic Recombinants Can Arise via SDSA

#### Random spore assay.

As discussed above, the ends-apart system is designed to test the possibility that NCO recombinants result from SDSA. We find that His^+^Leu^+^ double prototrophs formed by the ends-apart system have both predicted properties of the SDSA mechanism (Table 2). First, a significant fraction of His^+^Leu^+^ NCO recombinants should be geneticin-sensitive (Gen^S^), lacking the *KanMX* cassette (Figure 3A and 3B). We observed that 26% of NCO His^+^Leu^+^ recombinants carrying *R*-derived markers were Gen^S^. Second, the yield of the His^+^Leu^+^Gen^S^ class should be significantly lower in the construct lacking the duplicated segment. The yield of that was 2.4-fold lower in *R/D*::*KanMX* as compared with *R/D*::*KanMX*-*dup*. These findings provide evidence that NCO recombinants form via SDSA during meiosis.

**Table 2:**
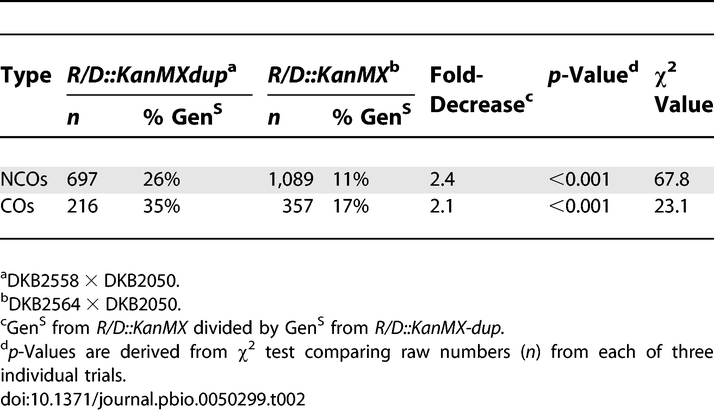
Geneticin Resistance Phenotype among His^+^Leu^+^ Recombinants

One departure from expectation was the recovery of residual His^+^Leu^+^Gen^S^ recombinants from *R/D::KanMX*. Because *R/D::KanMX* lacks the duplicated region of 5′-*leu2* sequences required for annealing of ends that are not extended through the *KanMX* gene, we predicted that it would not be capable of forming His^+^Leu^+^Gen^S^ products at appreciable frequency. However, 8.5% of His^+^Leu^+^ NCO recombinants produced by *R/D::KanMX* were Gen^S^. Sequence analysis of the repaired break site was performed to rule out DNA end-joining as a contributing mechanism, because end-joining usually results in deletion of nucleotides adjacent to the site of the break. A sample of these products (*n* = 20) showed sequences identical to the *R* parent and none contained deletions, thus eliminating the possibility that the unexpected class results from DNA end-joining. An explanation for the origin of the unexpected products is that, after receiving sequences from a homolog required to form a prototroph, dissociated ends can go on to invade the sister chromatid. End extension from a sister template would add a region, allowing the twice-extended end to anneal with the partner end or re-invade the homolog to acquire additional sequences. Although interhomolog recombination is the predominant meiotic mechanism, intersister recombination can also occur at a substantial frequency [17,50,51]. In addition, evidence for multiple rounds of invasion by the same end has been obtained for DSB-induced mitotic recombination in *Drosophila* and budding yeast [52,53]. Thus, we suggest that this unexpected class of recombinants results from at least two rounds of invasion with the first round being a homolog invasion and the second a sister invasion. Additional studies are necessary to test this mechanism.

Although only 26% of NCO recombinants recovered in this system fell into the class designed to be diagnostic for SDSA (His^+^Leu^+^Gen^S^), the data do not exclude the possibility that all NCOs—Gen^S^ and Gen^R^ —form by this mechanism. Gen^S^ and Gen^R^ NCOs may differ only in the degree of end extension prior to joint disruption and SDSA (Figure 3). On the other hand, the data are also consistent with the possibility that a significant subset of NCO events results from dHJ intermediates. The possibility that a fraction of NCOs are produced by dHJ intermediates is also consistent with earlier observations, where dHJs destined to form NCOs may have gone undetected as a result of being unstable or short-lived [20,21,26,27]. Furthermore, the patterns of heteroduplex DNA, while not easily reconciled with the possibility that all NCO recombinants arise via dHJs, are quite compatible with the possibility that a subset do [20–22,24,54]. Further studies will be needed to determine the fraction of NCOs resulting from SDSA and whether this fraction differs at different loci.

Previous studies using related systems to study mitotic SDSA provided evidence that extended ends can, after displacement, reinvade or anneal out of register such that triplications are formed ([33,45]; Figure S2). We examined 190 recombinants (90 His^+^Leu^+^Gen^R^ recombinants and 100 Arg^+^Ura^+^Gen^R^ recombinants) for evidence of triplications and found none (unpublished data). These results suggest that if triplications do occur, they do so at a frequency of less than 1.6% (assuming a binomial distribution of events). This finding indicates that re-invasion of a homologous chromatid and/or out-of-register reinvasion is relatively rare during meiosis.

#### Tetrad analysis.

Another test of the hypothesis that NCOs can arise via SDSA comes from measurement of the frequency of gene conversion of the *KanMX* heterology by tetrad analysis. The SDSA mechanism predicts that the duplicated 5′ segment on the donor in *R/D*::*KanMX-dup* should make it possible for a DSB at site 2 on the *R* chromatid to be repaired by SDSA without conversion of the *R* chromatid to *KanMX^+^* as illustrated in the leftmost column of Figure 3. Tetrads arising from the *R/D*::*KanMX-dup* strain were therefore predicted to show fewer 3Gen^R^:1Gen^S^ segregations than were those from *R/D::KanMX.* This prediction was fulfilled; there were significantly fewer (χ^2^ test, *p* = 0.01) 3:1 gene conversions among in *R/D::KanMX-dup*-derived tetrads (15/1,163, or 1.3%) as compared to *R/D::KanMX*-derived tetrads (27/933, or 2.9%) (Table 3). We further predicted that the duplicated segment should have no effect on 1Gen^R^:3Gen^S^ segregations. This is because 1Gen^R^:3Gen^S^ segregations arise from DSBs on the *D* chromatid, and thus repair events are expected to be templated by the *R* chromosome, which is identical in *R/D::KanMX-dup* and *R/D::KanMX*. This prediction was also fulfilled. These results are important because they provide evidence that the duplication has the predicted effect on conversion frequency in a situation that does not require selection of a specific subpopulation of spores. The random spore selection, while isolating diagnostic events, has the disadvantage that only the small subset of total conversion events with conversion tracts ending in certain intervals satisfies the selection criteria. The tetrad results mitigate concern that the selection method yields a nonrepresentative subset of events and supports the conclusion that a substantial fraction of conversion events result from SDSA.

**Table 3:**
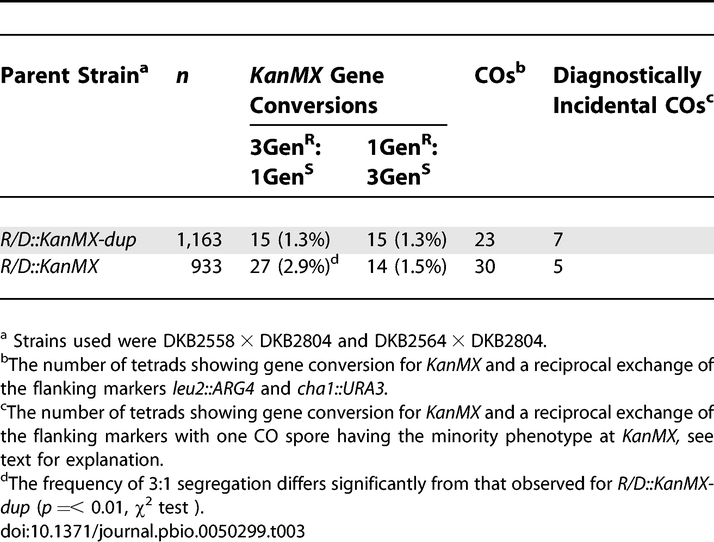
Tetrad Analysis for Unselected Tetrads

### COs in the Ends-Apart System

**Figure 4:**
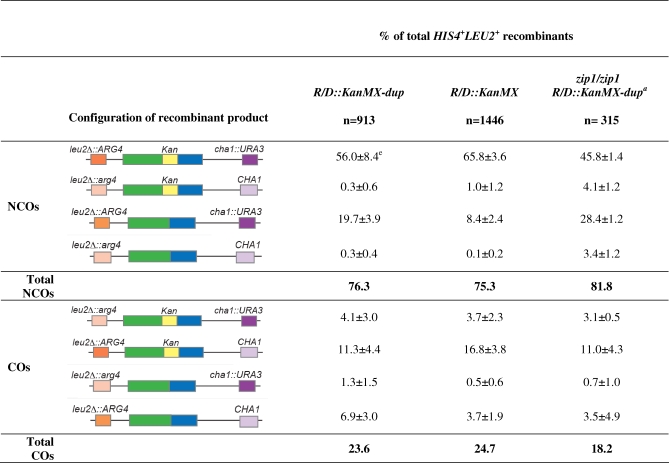
Genotypes of *HIS4^+^LEU2^+^* Recombinants The numbers given are percentages of double prototrophs with the configuration of markers indicated in the diagram on the left. The *R/D::Kan-MX-dup* strain was from DKB2558 × DKB2050; *R/D::Kan-MX* from DKB2564 × DKB2050; and *zip1/zip1 R/D::Kan-MX-dup* strain from DKB2379 × DKB2983. Experiments were performed on at least three separate cultures.

The ends-apart system is not designed to select CO recombinants that form by the canonical dHJ mechanism. This is because that mechanism yields pairs of conversion tracts in “trans,” meaning that one tract ends up on each of the two recombinant chromatids, rather than both ending up on the same chromatid. Because pairs of recombinant chromatids segregate to different spores, the selection for His^+^Leu^+^ double prototrophy is not expected to reveal the subset of events formed through dHJs. The “early decision model” for NCOs predicts that NCOs are more likely than COs to arise by a non-dHJ mechanism [25,26]. Given these considerations, the level of associated COs was expected to be much lower among double prototrophs than among single prototrophs. The observed total frequency of COs was about 60% among single prototrophs (Figure S1) and 24% among double prototrophs (Figure 4). This difference is statistically significant (*p* < 10^−40^).

Given that the canonical dHJ model does not readily account for the association of double-prototroph formation with the formation of a CO (as illustrated in Figure 3), how can the 24% of double prototrophs with CO configurations of markers be explained? There are two possible sources of COs; those that form as a result of the same event that forms the double prototroph and those that form in an incidental event. The frequency of incidental COs can be estimated from the *KanMX* gene conversion data in Table 3. In a tetrad exhibiting gene conversion, incidental COs are detected when a spore has both the CO configuration of flanking markers and the minority genotype at the converted locus (e.g., the Gen^S^ spore in a 3Gen^R^:1Gen^S^ tetrad). This diagnostic class for incidental COs represents one-half the total number of incidental exchanges. Pooling the data from two tetrad experiments, we found 17% (12 of 71) of conversions were diagnostic for incidental exchange and thus, approximately 34% of conversion tetrads have an incidental exchange. Incidental exchanges will alter the genotype of the spore containing a converted chromatid in 50% of events. Using the binomial distribution to obtain 95% confidence intervals, we estimate incidental exchanges in this system to alter the genotype of 9% to 25% of random spores**.** This implies that 43% ± 8% of events forming single prototrophs, but only 7% ± 8% of events forming double prototrophs are associated with a CO.**** Thus, many, if not all, of the CO events observed among double prototrophs are incidental. This result implies that double-prototroph selection strongly enriches for NCO recombinants as expected if NCOs form via SDSA. Any COs that do form in association with double prototrophs are likely to do so by a noncanonical mechanism such as the “strand-displacement–mediated” crossing over mechanism proposed by Allers and Lichten [31] (Figure S3).

To further characterize COs in this system, we examined the role of *ZIP1* on the array of double-prototroph genotypes. As mentioned previously, *ZIP1* is one of several genes required for normal levels of CO recombinants. At 30 °C, the temperature at which these experiments were performed, *zip1* reduces the frequency of COs from 1.4- to 4.8-fold in an interval-dependent manner [28,55]. A *zip1/zip1* mutant derivative of *R/D*::*KanMX-dup* was tested and found to show a modest (1.3-fold) but significant (*p* = 0.049) reduction in the level of CO recombinants among double prototrophs as compared to the *ZIP1^+^* strain (Figure 4). Most notably, the diagnostic class for SDSA (His^+^Leu^+^Gen^S^) among double prototrophs was 1.4-fold higher in *zip1* than in the wild-type control (*p* = 0.002). This finding is as expected if the frequency of SDSA events increases when CO formation is blocked.

### Elimination of Alternative Explanations

Three alternative explanations for the appearance of the diagnostic His^+^Leu^+^Gen^S^ recombinants were eliminated by additional experiments. First, because the duplication in *D::KanMX-dup* is a direct repeat flanking the heterologous *KanMX* insert, we considered the possibility that the diagnostic class of products could arise by intrachromatid single-strand annealing or “pop-out” type recombination. To address this possibility, we created an *R/D*::*KanMX-dup* diploid in which both copies of chromosome III contained the *KanMX* insertion at the break site between the *his4* and *leu2* heteroalleles. The diploid was allowed to sporulate, and His^+^Leu^+^ double prototrophs were selected. Examination of 500 single spores for the loss of geneticin resistance showed that all 500 spores had retained the *KanMX* insert, eliminating the possibility of a significant contribution from intrachromatid events.

Second, we considered that the diagnostic class of spores could be disomic in chromosome III, with one chromosome carrying *HIS4^+^* and the other *LEU2^+^*. CHEF gel analysis was performed on 39 His^+^Leu^+^Gen^S^ spores, 15 from *R/D*::*KanMX-dup*, and 24 from *R/D*::*KanMX*. No evidence of disomy was found in the products from either of the parental diploids (unpublished data).

Last, an alternative scenario compatible with the canonical dHJ resolution model would invoke the formation of a large single-stranded loop in the heteroduplex DNA as an intermediate in the formation of the diagnostic His^+^Leu^+^Gen^S^ recombinants. This could occur if a heteroduplex tract forms with one end between the two markers in *his4* and the other end between the two markers in *leu2*. In this case, the *KanMX* region would form a large single-stranded loop. Previous studies have shown that such large loops can form and are repaired during meiosis [56,57]. However, two considerations make it highly unlikely that loop repair accounts for His^+^Leu^+^Gen^S^ recombinants. Because essentially all NCO recombinants recovered are *R* chromatids, a loop repair scenario would have to involve three correction events using alternating templates: *D* (at *his4B*)*, R* at (at the *KanMX* insertion site)*,* and *D* (at *leu2K*). If loop repair were the source of the Gen^S^ recombinant class, we would expect the total yield of double prototrophs from the parental strain not containing the *KanMX* insertion (the *R/D* control strain) to be much higher than that from the strains containing it, because a single continuous tract could give rise to that class. A second consideration is that the construct was specifically designed such that the vast majority of recombinogenic DSBs would be directly opposite the *KanMX* heterology, and not between the two *his4* heteroallelic loci or the two *leu2* heteroallelic loci.

### Double Prototrophs Result from a Single Recombination Event

For the ends-apart system to provide evidence for SDSA, the properties of the system must reflect the expectation that the majority of double-prototrophic recombinants result from a single site 2 DSB on the *R* chromatid. The following considerations show that the system meets this requirement.

The first line of evidence indicating that double prototrophs result from a single DSB is provided by analysis of the flanking markers *ARG4* and *URA3*. The formation of prototrophs from mutant heteroallele pairs occurs mainly via gene conversion of one of the two markers. This expectation has been confirmed for the single prototrophs formed by both the *his4X/his4B* and the *leu2K/leu2R* heteroallele pairs used here (unpublished data). The established properties of gene conversion indicate that formation of a functional allele from mutant heteroalleles occurs predominantly via conversion tracts that extend from a DSB site past the break-proximal, but not the break-distal mutation [58,59]. Assuming that most conversion tracts yielding prototrophy are of this type, the configuration of mutations in *R/D* heterozygotes dictates that a single DSB is far more likely to give rise to a double prototroph if it occurs on the *R* rather than the *D* chromosome. Flanking markers allow unambiguous identification of the chromatid that initiated the formation of NCO double prototrophs. Importantly, almost all NCO double prototrophs had flanking markers from the *R* chromosome (99% for *R/D*::*KanMX-dup* and 98% for *R/D*::*KanMX,* Figure 4). This result indicates that the events that form *HIS4^+^* alleles in double prototrophs initiate to the right of *his4B* on *R* (as drawn in Figure 2) and those forming *LEU2^+^* alleles initiate to the left of *leu2K* on *R*. Therefore, the vast majority of NCO double-prototrophic recombinants are explained by a single initiation event on *R*, located between the *his4B* and *leu2K* markers (Text S1).

A second line of evidence supporting the assumption that the majority of double prototrophs result from a single DSB is provided by estimates of double-prototroph event frequency derived under the converse prediction. The converse prediction is that the double prototrophs result from two independent events, each of which yields a single prototroph. This analysis takes into account the frequency at which single prototrophs form, as well as information provided by the flanking markers. As mentioned above, nearly all NCO recombinants have *R-*flanking markers. This finding places a constraint on which types of single prototroph–generating events are capable of combining to yield the observed genotypes of NCO double prototrophs. The simplest contribution to the double-prototroph class would be the combination of events that both give the recipient-derived NCO (R-NCO) configuration of flanking markers (i.e., *ARG4^+^URA3^+^)* (Table S1, Equation 1).

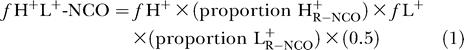



The product of the two relevant single- prototroph frequencies is multiplied by 0.5, because the two events are equally likely to initiate on either of two recipient chromatids, thus are only expected to involve the same recipient chromatid half the time. There are two additional sources of R-NCO recombinants resulting from the combination of two single-prototroph CO events. The first of these (Table S1, Equation 2) combines *ARG4^+^ HIS4^+^ ura3* and *arg4 LEU2^+^ URA3^+^* Cos, while the second (Table S1, Equation 3) combines *ARG4^+^ LEU2^+^ ura3* and *arg4 HIS4^+^ URA3^+^* COs.

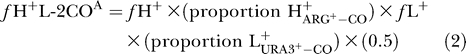


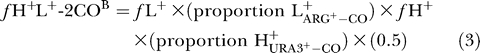



The sum of Equations 1, 2 and, 3 gives an estimate of the number of observed NCO double prototrophs that might have resulted from two independent events. This method gives a modest overestimate, because it does not take crossover interference into account. The analysis indicates that double independent events account for less than 10% of observed double-prototrophic NCO recombinants obtained from the relevant parent diploids.

Another approach to eliminating the possibility that double prototrophs result from two independent events is to reduce the frequency of recombination initiation and examine the effect on the frequency of double prototrophs. We used *spo11-D290A-HA3-HIS6::KanMX4* (hereafter referred to as *spo11D290A*), a leaky allele of *SPO11* [60]. *SPO11* encodes the transesterase responsible for forming meiotic DSBs. The leaky allele was shown in previous work to reduce meiotic recombination frequencies about 3-fold [61]. In our system, this allele reduced recombination frequencies to 29% of wild-type levels (3.4-fold decrease) for *HIS4^+^*, and 15% of wild-type levels (6.9-fold decrease) for *LEU2^+^*. In our ends-apart system, if double prototrophs arise from two independent breaks, then we would expect the double-prototroph frequency to be reduced in *spo11D290A* by the product of the two single-prototroph reductions (i.e.*,* 0.29 × 0.15 = 0.04). On the other hand, if double prototrophs arise only via single breaks, then we would expect the reduction of double-prototroph frequency to be the same as the reduction in single prototrophs (i.e.*,* reduced to between 29% and 15% of the level seen in *SPO11^+^*). As shown in Table 1, the *spo11D290A* mutation reduced double-prototroph formation to 20% of wild-type levels, well within the range of the effect on single prototrophs. In summary, we have shown three lines of evidence collectively showing that the vast majority of the selected double prototrophs arise from a single DSB between *his4B* and *leu2K*.

### The CO versus NCO Decision

Numerous observations point to the fact that COs and NCOs arise via a common intermediate. The hypothesis that dHJ resolution is the molecular process responsible for divergence of CO and NCO pathways in budding yeast had, until recently, been long-standing. However, a growing body of evidence that commitment to the CO pathway occurs before the stage when dHJs form has been mounting [15,26,27,62]. The results presented here provide evidence that NCO recombinants can result from the ejection of extended 3′ ends from joint molecules and subsequent annealing. If most, or all, NCOs do result from SDSA and most COs from HJs, then what is responsible for determining whether a recombination event will become a CO or an NCO? In addressing this question, it is important to distinguish “commitment” to a particular pathway from execution of the first detectable molecular event at which the two pathways diverge. Commitment may occur at a recombination stage when methods available for assaying intermediates do not distinguish the two pathways. The critical event for executing the CO/NCO decision could be the loading of a helicase at a heteroduplex joint. Previous studies have shown that helicases can act to enhance or reduce the ratio of CO to NCO recombinants. The first intermediate appearing to be CO pathway-specific is one in which only a single end is stably engaged with the donor duplex [15]. This single end invasion (SEI) intermediate is converted to a ligated dHJ, which in turn is resolved to a CO [15,26,27]. Formation of both SEIs and CO recombinants depends on Mer3. Mer3 is a branch-specific helicase that appears to stabilize single-end intermediates by increasing the hybrid tract lengths [27,63,64]. A different helicase may be required to disrupt intermediates once 3′ end extension has occurred, allowing progression through the NCO pathway. One candidate for a joint disruption helicase is Sgs1 [65,66]. A modest enhancement of CO frequency can been seen in *sgs1* mutants [67–69], consistent with a role in promoting SDSA. However, if Sgs1 does promote NCOs, there must be an efficient alternative pathway to NCOs that operates in its absence, as *sgs1* mutants still show high levels of NCOs. It is also worth noting that end-extension during SDSA may involve replication-driven bubble migration [70]. In this case, joints may dissociate without the aid of a helicase.

Although joint disruption could be the first detectable step in distinguishing the two pathways, this step must follow the stage at which sites are designated to follow one path or the other; mechanisms that dictate the nonrandom distribution of meiotic COs appear to act prior to the actual disruption or ligation of homologous joints (reviewed in [25]).

The data presented here provide evidence that a major fraction of NCO recombinants in budding yeast result from SDSA rather than from dHJ-mediated recombination. Future studies will be required to determine the relative contribution of SDSA and dHJs to NCO recombinants. It will also be of interest to learn if the prevalence of SDSA varies between genetic loci and/or between species.

## Materials and Methods

### Yeast strains.

All strains (Table S2) are isogenic heterothallic derivatives of the S. cerevisiae strain SK-1 [71]. Yeast strains used in all experiments were constructed by standard genetic crosses, or by LiAc transformation [72]. Previously described conditions were used for growing and maintaining strains [12]. All strains contain the synthetic recombination hotspot *HIS4::LEU2* [11]. This hotspot construct contains a copy of the *LEU2* gene inserted centromere-distal to the *HIS4* coding region. The relevant mutant heteroalleles of these two genes were created by restriction digest fill-ins: *his4B* and *his4X* heteroalleles were generated from *Bgl*II and *Xho*I sites, respectively [46], and *leu2K* and *leu2R* were generated from *Kpn*I and *Eco*RI sites, respectively [73]. The experimental strains *D::KanMX* (DKB2564) and *D::KanMX-dup* (DKB2558) were created by transformation of the *his4X::leu2R*-containing control strain (DKB2562) with a PCR-amplified fragment of the G418-resistance *KanMX2* cassette [47]. Primers for the amplification (Text S2) contained targeting tails homologous to the regions up- and downstream of DSB site 2, which is located approximately 500 bases upstream of the *HIS4::LEU2* junction [11]. The targeting tails were engineered such that, upon transformation, 300 bases of sequence would be duplicated in *D::KanMX-dup* (DKB2558) and not in *D::KanMX* (DKB2564). This was achieved by taking advantage of the fact that DNA fragments can recombine into the yeast genome in an “ends-in” or “ends-out” configuration [74]. *D::KanMX* was constructed using the ends-out targeting tails, creating a disruption insertion directly between the two continuous target sequences. *D::KanMX-dup* was constructed using the ends-in targeting tails, which invade target sequences separated by 300 bp; this targeting reaction will “gap repair” across the 300-bp region and recombine into the genome, thereby duplicating that region.

All parental strains contain a complete deletion of all coding sequence from the *LEU2* locus, located 23 kb centromere-proximal from the hotspot. The deleted locus is marked either with *ARG4* or *arg4*. The *leu2Δ::ARG4* allele was created by transforming a *LEU2* strain with a PCR-amplified copy of the *ARG4* gene, using primers with 40-bp tails of terminal homology to regions directly up- and downstream of the *LEU2* open reading frame. Chromosomes designated *R* carry *leu2Δ::ARG4.* A derivative of *leu2Δ::ARG4*, designated *leu2Δ::arg4*, was generated by isolation of ectopic recombinants among random spores derived from a *leu2Δ::ARG4/leu2Δ::ARG4 arg4/arg4* diploid strain. Recipient strains are marked by insertion of a 0.8-kb fragment containing the *URA3* gene located 40 kb centromere-distal from the hotspot. Donor strains lack this insertion.

An SK-1 derivative containing the *spo11-D290A-HA3-HIS6::KanMX4* allele [61] was generously provided by S. Keeney and introduced into strains carrying *D* and *R* chromosomes by conventional genetic crosses.

### Random spore analysis.

Random spore analysis of recombination in strains containing heterozygous chromosome markers was carried out by a method designed to minimize any contribution by mitotic recombination to the population of selected recombinants. Diploid strains examined were created at the time of each experiment by first isolating single colonies of parental haploid strains on YPDA plates. Assays were performed in triplicate by selecting three single colonies from each strain. These were grown in large patches on YPDA plates for 12 h, or 24 h for *spo11-D290A-HA3-HIS6::KanMX4-*containing strains. Parental haploids were then mated on fresh YPDA plates for 8 h. After 8 h, the mating patches containing newly-formed diploids were scraped off the plates and suspended in 50 ml liquid SPS at an optical density at 600 nm (OD_600_) of 0.5. This suspension was incubated at 30 °C, with shaking, until the culture reached an OD_600_ of 0.9, approximately 5 h later.

Meiosis was induced by shaking the cells in 50 ml SPM+1/5 COM liquid sporulation medium at 30 °C for 18–24 h. Ascus walls were digested for 3 h at 37 °C with 20 μg/ml zymolyase 100-T and 0.4% β-mercaptoethanol. Three volumes of NP-40 lysis buffer (0.02% (v/v) NP-40, 50 mM Tris, 150 mM NaCl, 2 mM EDTA) was added and incubated at room temperature for 30 min. A suspension of single spores was then generated by sonication. Serial dilutions were made to 10^−6^, and spores were plated on both complete and selective media (i.e., plates lacking histidine, leucine, or both). After 3 d growth, recombination frequencies were calculated according to the following formulae*: f*(His^+^) = #His^+^/#Com, *f*(Leu^+^) = #Leu^+^/#Com, and *f*(H^+^L^+^) = #His^+^Leu^+^/#Com, where “#Com” indicates the number of spores growing on complete medium, representative of the entire population of viable spores.

### Tetrad analysis.

For tetrad analysis, recipient parental strains were modified to include additional heterozygous markers, *ade2* and *lys2,* to avoid the chance that a 3:1 segregation of *KanMX* resulted from a false tetrad. All 3:1 segregations of *KanMX* showed 2:2 segregation for both *ade2* and *lys2*. Parent haploids were mated and allowed to sporulate immediately, as above. Tetrads were treated with 500 μg/ml zymolyase 100-T for 5 min at 37 °C before dissection using a conventional micromanipulator (Zeiss).

## Supporting Information

Figure S1 Genotypes of Single-Prototroph Recombinants (671 KB DOC)

Figure S2 Triplications Formed by a Second, Out-of-Register Invasion of an Extended End (150 KB DOC)

Figure S3 Strand-Displacement–Mediated Crossing Over (60 KB PDF)

Table S1 Expected Values of Double-Prototroph Formation, Given Two Independent Events (34 KB DOC)

Table S2 Yeast Strains Used in This Study (36 KB DOC)

Text S1 Comparison of the Frequencies of NCO Single and Double Prototrophs under the Assumption That all Prototrophs Result from a Single DSB (43 KB DOC)

Text S2 Primers to Amplify *KanMX* Cassette and Create *D::KanMX* and *D::KanMX-dup* Constructs (21 KB DOC)

## Abbreviations

CO: crossover
*D*: donor
dHJ: double Holliday junction
DSB: double-strand break
Gen^R^: geneticin resistant
Gen^S^: geneticin sensitive
HJ: Holliday junction
NCO: noncrossover
*R*: recipient
SDSA: synthesis-dependent strand annealing
