# Supplementary material for: Synthesis-Dependent Strand Annealing in Meiosis
Source: PLoS Biol. 2007 Nov 6;5(11):e299. doi: 10.1371/journal.pbio.0050299 (PMC2062477; doi:10.1371/journal.pbio.0050299)

Figure S3. Strand-Displacement-Mediated Crossing Over

A) The *R* chromatid is cleaved at Site 2 and 5’ ends are resected, leaving 3’ overhanging tails. B) Invasion of an end forms a D-loop and the invading end is extended to include the duplicated segment. C) The D-loop migrates away from the site of the DSB and in doing so is converted to a dHJ. Migration also displaces the extended end. D) The extended and displaced end anneals to its partner. E) Repair synthesis and dHJ resolution yields a downstream crossover.

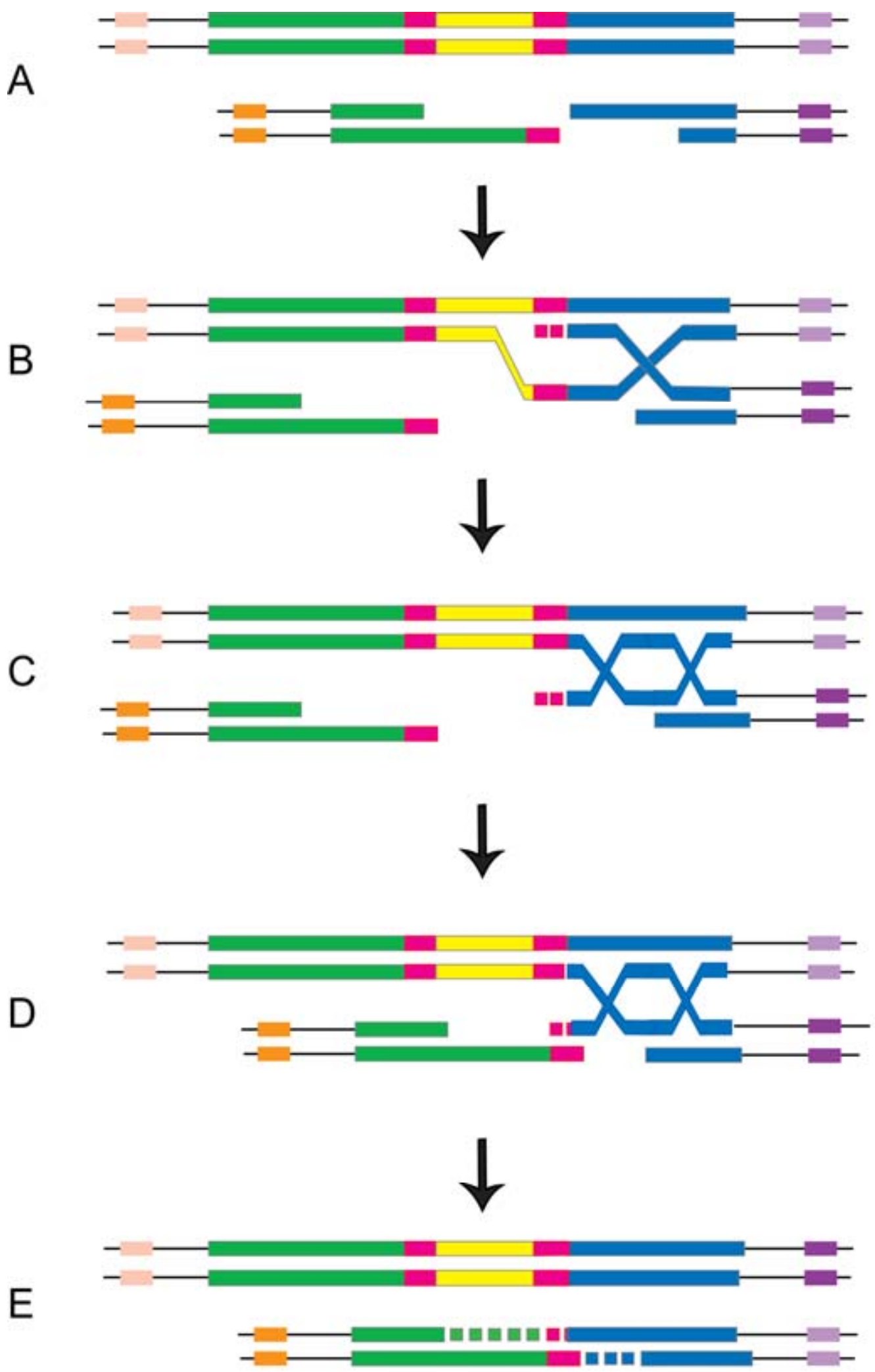

Supplement: Supplementary file 1 [file pbio.0050299.sg003.pdf]
