# Supplementary material for: Synthesis-Dependent Strand Annealing in Meiosis
Source: PLoS Biol. 2007 Nov 6;5(11):e299. doi: 10.1371/journal.pbio.0050299 (PMC2062477; doi:10.1371/journal.pbio.0050299)
Supplement: Supplementary file 2 [file pbio.0050299.sd001.doc]

**Text S1. Comparison of the frequencies of NCO single and double prototrophs under the assumption that all prototrophs result from a single DSB**

We argue in the main manuscript that single prototroph frequencies do not account for the frequency of double prototrophs under the assumption that double prototrophs arise from two independent events. How do single and double prototroph frequencies compare under the assumption of a single DSB? If we assume that all recombination events yielding NCO prototrophs on the *R* chromatid result from a single DSB at Site 2, it is possible to use single prototroph frequencies to predict double prototroph frequencies. In this analysis, a prediction of double prototroph frequency can be obtained by first calculating the efficiency with which a DSB yields each type of single prototroph. The product of the two efficiencies is then multiplied with the DSB frequency.

Analysis of the *his4::leu2* break site in a *rad50S* mutant background, where DSB ends are neither resected nor repaired after a break, showed that 3% of DNA suffers a break at this site ([42]; our unpublished data). Therefore, 1.5% of DNA will have suffered a break on the recipient chromosome, *i.e.,* 1.5% of spores should contain a recipient chromosome that suffered a Site 2 break. Using numbers from the *R/D*::*KanMX-dup* strain as an example, the expected frequency of double prototrophs can be estimated using the DSB frequency (ƒ_DSB_) as follows:

*f*His^+^Leu^+^_NCO-R-EXP_ = (*f*His^+^_NCO-R_/ *f*_DSB_)*(*f*Leu^+^_NCO-R_/ *f*_DSB_)***** *f*_DSB_

=*f*His^+^_NCO-R_**f*Leu^+^_NCO-R_**f*_DSB_^-1^

where *f*His^+^_NCO-R_ is the product of the total frequency of His^+^ prototrophs and the proportion of His^+^ prototrophs that are NCOs with the R configuration of flanking markers; and ƒLeu^+^_NCO-R_ is the product of the total frequency of Leu^+^ prototrophs and the proportion of Leu^+^ prototrophs that are NCOs with the *R* configuration of flanks. For the *R/D*::*KanMX-dup* data:

*f*His^+^Leu^+^_EXP_ = (4.6 x 10^-3^)*(0.239)*( 1 x 10^-2^)*(0.251)*(0.015)^-1^= 1.8x10^-4^.

The frequency of observed NCO double prototrophs (*f*His^+^Leu^+^_OBS_) is the product of total frequency of double prototrophs and the NCO fraction. For the *R/D*::*KanMX-dup* data:

*f*His^+^Leu^+^_OBS_ = 8.9 X 10^-5^ *0.75 = 6.7x10^-5^.

Thus, the observed frequency of double prototrophs is 2.7 fold lower than that predicted by this method. Previous studies provide two non-mutually exclusive explanations for this outcome. First, tetrad analysis provides evidence for asymmetry of heteroduplex tract lengths [20,21,23]. If bi-directional heteroduplex tracts tend to be longer on one side than on the other, then the frequency with which a single DSB gives a double prototroph will be reduced relative to that predicted by the method above. Second, our method makes the assumption that all NCO events involve bidirectional tracts on a single chromatid. If some NCOs form by the canonical dHJ mechanism, then this too will lower the yield of double prototroph relative to that predicted by this method. Results of previous studies indicate that it is likely that both tract length asymmetry and events involving tracts on two chromatids contribute to the observed ratios of single to double prototrophs.
