# Supplementary material for: Synthesis-Dependent Strand Annealing in Meiosis
Source: PLoS Biol. 2007 Nov 6;5(11):e299. doi: 10.1371/journal.pbio.0050299 (PMC2062477; doi:10.1371/journal.pbio.0050299)
Supplement: Supplementary file 3 [file pbio.0050299.sd002.doc]

**Text S2. Primers to amplify *KanMX* cassette and create *D::KanMX* and *D::KanMX-dup* constructs**

After amplifying the *KanMX* cassette, the primers used carry tails that are targeted to the genome. Primer sequences are as follows:

Primer 1: 5'CAAAATCAATTGTCCTGTACTTCCTTGTTCATGTGTCTTCcgcgttggccgattcat3'

Primer 2: 5'TTTAGATGACTATTTCTCATCATTTGCGTCATCTTCTAAacacatacgatttaggtgacac3'

Primer 3: 5'AGAAATAGAGTATAATTATCCTATAAATATAACGTTTTTGacacatacgatttaggtgacac3’

Capital letters denote the targeting "tail" and small letters denote the portion designed to amplify the *KanMX* cassette. Primers 1+2 were used in creating *D::KanMX-dup*, and primers 1+3 were used in creating *D::KanMX*.
