# Supplementary material for: Synthesis-Dependent Strand Annealing in Meiosis
Source: PLoS Biol. 2007 Nov 6;5(11):e299. doi: 10.1371/journal.pbio.0050299 (PMC2062477; doi:10.1371/journal.pbio.0050299)
Supplement: Supplementary file 4 [file pbio.0050299.sg001.doc]

**Figure S1. Genotypes of single prototroph recombinants**

Experiments were performed on three separate days, with three independent cultures of each strain, with the exception of Leu^+^ spores from R/*D::KanMX-dup,* where spores from one day (three independent cultures) were scored. The repeats within each day were not found to be significantly different from each other, and therefore were pooled. The numbers given are percentages of double prototrophs with the configuration of markers indicated in the diagram on the left. The *R/D::Kan-MX-dup* strain was from DKB2558 x DKB2050; *R/D::Kan-MX* from DKB2564 x DKB2050.

|  |  | ***% of HIS4^+^* recombinants** | | ***% of LEU2^+^* recombinants** | |
| --- | --- | --- | --- | --- | --- |
|  | **Configuration of recombinant product** | ***R/D::KanMX-dup***  **n=432** | ***R/D::KanMX***  **n=442** | ***R/D::KanMX-dup***  **n=144** | ***R/D::KanMX***  **n=414** |
| **NCOs** |  | 14.6±5.0 | 17.5±1.4 | 6.3±2.1 | 3.4±0.3 |
|  |  | 15.7±3.3 | 12.9±3.4 | 13.2±1.2 | 15.2±1.4 |
|  |  | 9.3±0.4 | 11.8±2.0 | 18.8±2.1 | 20.8±0.4 |
|  |  | 0.0±0.8 | 0.1±0.2 | 1.4±1.2 | 1.0±0.2 |
| **Total NCOs** |  | **40.5** | **42.3** | **39.7** | **40.4** |
| **COs** |  | 3.9±1.1 | 4.3±0.4 | 41.7±2.1 | 37.0±1.7 |
|  |  | 48.8±5.4 | 48.8±6.9 | 0.7±1.2 | 3.0±0.7 |
|  |  | 1.2±0.4 | 0.5±0.8 | 5.6±2.4 | 2.3±1.0 |
|  |  | 5.6±3.0 | 4.1±2.1 | 12.5±4.2 | 17.3±0.9 |
| **Total COs** |  | **59.5** | **57.7** | **60.5** | **59.6** |
