# Supplementary material for: Synthesis-Dependent Strand Annealing in Meiosis
Source: PLoS Biol. 2007 Nov 6;5(11):e299. doi: 10.1371/journal.pbio.0050299 (PMC2062477; doi:10.1371/journal.pbio.0050299)
Supplement: Supplementary file 5 [file pbio.0050299.sg002.doc]

**Figure S2. Triplications formed by a second, out-of-register invasion of an extended end.**

Colors represent the same markers as described in Figure 2. A) The *R* chromatid is cleaved at Site 2 and 5’ ends are resected, leaving 3’ overhanging tails. B) An end invades the homolog, forming a D-loop. C) After the opportunity for mismatch correction, this invading end is extended through the *KanMX* insert and duplicated segments. D) The extended end disengages. E) The disengaged end reinvades at the originally-invaded location, the duplicated segment. F) Another round of extension creates another copy of the *KanMX* insert and copies the left-side marker. G) The twice-extended end disengages again and reanneals back to its partner end. H) Repair synthesis fills in the resected end on the right side to repair the molecule.
