# Supplementary material for: Synthesis-Dependent Strand Annealing in Meiosis
Source: PLoS Biol. 2007 Nov 6;5(11):e299. doi: 10.1371/journal.pbio.0050299 (PMC2062477; doi:10.1371/journal.pbio.0050299)
Supplement: Supplementary file 6 [file pbio.0050299.st001.doc]

Table S1. Expected values of double prototroph formation given two independent events

| **Strain** | ***R/D::control*** | ***R/D::KanMX-dup*** | ***R/D::KanMX*** |
| --- | --- | --- | --- |
| **Eq. 1** | (3.8x10^-3^)(0.23)(1.1x10^-2^) (0.36)(0.5) = 1.8x10^-6^ | (4.6x10^-3^)(0.24)(1.0x10^-2^) (0.25)(0.5) = 1.4x10^-6^ | (4.5x10^-3^)(0.29)(0.9x10^-2^) (0.24)(0.5) = 1.4x10^-6^ |
| **Eq. 2** | (3.8x10^-3^)(0.54)(1.1x10^-2^) (0.37)(0.5) = 4.4x10^-6^ | (4.6x10^-3^)(0.53)(1.0x10^-2^) (0.47)(0.5) = 6.0x10^-6^ | (4.5x10^-3^)(0.53)(0.9x10^-2^) (0.39)(0.5) = 4.2x10^-6^ |
| **Eq. 3** | (3.8x10^-3^)(0.05)(1.1x10^-2^) (0.20)(0.5) = 1.5x10^-7^ | (4.6x10^-3^)(0.05)(1.0x10^-2^) (0.13)(0.5) = 1.6x10^-7^ | (4.5x10^-3^)(0.05)(0.9x10^-2^) (0.13)(0.5) = 2.0x10^-7^ |
| **Total (Exp)^a^** | 6.4x10^-6^ | 7.5x10^-6^ | 5.8x10^-6^ |
| **Total (Obs)^b^** | 6.4x10^-5^ | 8.9x10^-5^ | 7.9x10^-5^ |
| **Obs/Exp^c^** | 10 | 12 | 14 |

^a^ The total value of expected double prototroph formation, resulting from adding the values derived from equations Eq. 1, Eq. 2, Eq. 3.

^b^ The total value of actual observed double prototroph formation.

^c^ The fold-increase of observed over expected double prototrophs.
