# Supplementary material for: Synthesis-Dependent Strand Annealing in Meiosis
Source: PLoS Biol. 2007 Nov 6;5(11):e299. doi: 10.1371/journal.pbio.0050299 (PMC2062477; doi:10.1371/journal.pbio.0050299)
Supplement: Supplementary file 7 [file pbio.0050299.st002.doc]

**Table S2. Yeast strains used in this study**

| **Strain** | **Genotype** |
| --- | --- |
| DKB2050 | *MATα ho::LYS2, lys2, ura3, leu2Δ::ARG4, arg4, his4B::leu2K, cha1::hisG-URA3-ade2(BglII-StuI)* |
| DKB2379 | *MATα ho::LYS2, lys2, ura3, leu2Δ::ARG4, arg4, his4B::leu2K, cha1::hisG-URA3-ade2 (BglII-Stu1), zip1::LYS2* |
| DKB2558 | *MAT***a** *ho::LYS2, lys2, ura3, leu2Δ::arg4, arg4, his4X::KanMX2::leu2R* |
| DKB2562 | *MAT***a** *ho::LYS2, lys2, ura3, leu2Δ::arg4, arg4, his4X::leu2R* |
| DKB2564 | *MAT***a** *ho::LYS2, lys2, ura3, leu2Δ::arg4, arg4, his4X::KanMX2::leu2R* |
| DKB2772 | *MAT***a** *ho::LYS2, lys2, ura3, leu2Δ::ARG4, arg4, his4B::leu2K, cha1::hisG-URA3-ade2 (BglII-Stu1), spo11-D290A-HA3-HIS6::KanMX4* |
| DKB2777 | *MAT***a** *ho::LYS2, lys2, ura3, leu2Δ::arg4, arg4, his4X::KanMX2::(5’-leu2)leu2R, spo11-D290A-HA3-HIS6::KanMX4* |
| DKB2798 | *MATα ho::LYS2, lys2, ura3, leu2Δ::arg4, arg4, his4B::KanMX2::(5’-leu2)leu2K* |
| DKB2803 | *MATα ho::hisG, lys2, ura3, leu2Δ::ARG4, ade2::LK, arg4, his4B::leu2K, cha1::hisG-URA3-ade2 (BglII-Stu1)* |
| DKB2850 | *MATα ho::LYS2, lys2, ura3, leu2Δ::ARG4, arg4, his4B::leu2K--URA3* |
| DKB2983 | *MAT***a** *ho::lys2, lys2, ura3, leu2Δ::arg4, arg4, his4X::KanMX2::(5’-leu2)leu2R, zip1::LYS2* |
| DKB2986 | *MATα ho::LYS2, lys2, ura3, trp1, leu2 ::ARG4, arg4, TRP1--his4B::leu2K--URA3* |
